# P2 Receptors in Cardiac Myocyte Pathophysiology and Mechanotransduction

**DOI:** 10.3390/ijms22010251

**Published:** 2020-12-29

**Authors:** Sun-Hee Woo, Tran Nguyet Trinh

**Affiliations:** Laboratory of Pathophysiology, College of Pharmacy, Chungnam National University, 99 Daehak-ro, Yuseong-gu, Daejeon 34134, Korea; tranctu1994@gmail.com

**Keywords:** cardiac myocyte function, P2X receptors, P2Y receptors, extracellular ATP, mechanical signaling, pathohysiological roles

## Abstract

ATP is a major energy source in the mammalian cells, but it is an extracellular chemical messenger acting on P2 purinergic receptors. A line of evidence has shown that ATP is released from many different types of cells including neurons, endothelial cells, and muscle cells. In this review, we described the distribution of P2 receptor subtypes in the cardiac cells and their physiological and pathological roles in the heart. So far, the effects of external application of ATP or its analogues, and those of UTP on cardiac contractility and rhythm have been reported. In addition, specific genetic alterations and pharmacological agonists and antagonists have been adopted to discover specific roles of P2 receptor subtypes including P2X4-, P2X7-, P2Y2- and P2Y6-receptors in cardiac cells under physiological and pathological conditions. Accumulated data suggest that P2X4 receptors may play a beneficial role in cardiac muscle function, and that P2Y2- and P2Y6-receptors can induce cardiac fibrosis. Recent evidence further demonstrates P2Y1 receptor and P2X4 receptor as important mechanical signaling molecules to alter membrane potential and Ca^2+^ signaling in atrial myocytes and their uneven expression profile between right and left atrium.

## 1. Introduction

ATP has long been recognized as an intracellular energy source. ATP is now widely accepted as a key extracellular chemical messenger released from many cell types including neuronal cells, endothelial cells, muscle cells, and it significantly regulates different cell functions via P2 purinergic receptors [1]. Extracellular ATP exerts several important effects in cardiac myocytes, such as negative and positive inotropic effects, negative or positive chronotropic effects as well as antihypertrophic effects [2]. It is also known that ATP inhibits glucose transport in the heart [3]. Cardiac cells from different heart regions and from different species have different contexts of purinergic receptor subtypes. Such context of P2 receptor subtypes appears to determine ATP-mediated cellular responses, such as inotropy and chronotrophy. Use of transgenic and knock-out animals as well as pharmacological agonists and antagonists enabled understanding of specific function of each P2 receptor subtype in the heart under physiological and pathological conditions. The present review concentrates on the effects of ATP on cardiac functions at the cellular levels and whole heart under physiological and pathological conditions and recent advances in discovering role of certain P2 receptors in atrial myocytes in mechanotransduction and Ca^2+^ regulation.

## 2. ATP as an Extracellular Chemical Messenger

Early studies have demonstrated ATP exocytosis using new bioluminescence methods with cell surface attached firefly luciferase [4,5], or atomic force microscopy [6]. Cellular ATP release can be detected by luciferin-luciferase assay at the multicellular levels [7,8,9], and also by reporter cells at the single cell level [10,11]. Using a reporter cell expressing a P2X receptor or P2Y receptor one can measure ATP release from nearby single target cells in real-time as P2X receptor currents or as P2Y receptor-mediated cytosolic Ca^2+^ increase that are activated by ATP [10,11,12,13]. In the mammalian cells there are multiple pathways of ATP release from intracellular space to external space. They include gap junction channels, connexins [9,11,14] and pannexins [8], cystic fibrosis transmembrane conductance regulator-linked pathway [15,16,17,18,19,20,21], maxi anion channels [12], volume-regulated Cl^−^ channel [22], and exocytosis [23,24]. The ATP release processes in the cells are regulated by different types of stimuli including mechanical stimuli [9,25,26]. A line of evidence strongly support that ATP is a co-neurotransmitter in sympathetic nerves around the blood vessel [27]. In the atrial myocardium of the human heart, the nerve terminal varicosities form a dense network innervating the cardiac muscle, coming into close apposition with the cardiac myocytes [28]. In addition to neuron [29,30,31], cardiac myocytes [8,9,11,32,33,34,35], endothelial cells [36,37,38], smooth muscle cells [36,39,40], platelets [41,42,43], and other cell types have been shown to release ATP from cytosol to extracellular space under a stimulus via one or two type(s) of ATP release pathways.

Extracellular ATP concentrations are thought to be about 1–40 nM and intracellular ATP concentrations are about 10 mM. In the coronary artery in the heart, the levels of ATP are physiologically very low (1 nM; [44]), mainly because ATP is rapidly degraded to ADP, AMP and adenosine by soluble and membrane bound ectonucleotidases (ecto-ATPases) [45,46]. However, in the interstitial fluid in the heart higher levels (40 nM) of ATP can be measured [47]. ATP level in the coronary artery in the heart significantly increases under electrical stimulation, application of cardiotonic agents [44,48,49,50], mechanical stretch [51], higher blood flow [48,52], high workload [53] and hypoxia/ischemia [32,33,34,35,41,47,50]. In addition, a line of evidence shows significant ATP release from cardiac ventricular and atrial myocytes under mechanical stresses. Stretch has been demonstrated using luciferin-luciferase assay to induce ATP release from ventricular myocytes of mouse heart through the pannexin-1 [7] and from atrial cells via pannexin-2 gap junction channels [8]. Recently, it has been shown using P2X7 receptor-expressing human embryonic kidney (HEK) 293 cells as a reporter that shear stress elicits immediate ATP release from isolated adult rat atrial myocytes, thereby inducing two different types of global Ca^2+^ waves [11]. It has also been shown that left atrial (LA) cells release ATP more than right atrial (RA) cells under the same shear force, and that the generations of different types of Ca^2+^ waves depend on P2 receptor subtypes (see below; [9,11]). Atrial ATP release under shear stress is known to be mediated by connexin 43 (Cx43) hemichannels [9].

## 3. P2 Receptors in Cardiac Muscle and Their Pharmacological Properties

Extracellular ATP and its analogs initiate large effects via cell surface P2 purinergic receptors in the cardiovascular system [1,2,53,54,55,56,57]. The P2 purinergic receptors are further divided in P2X ionotropic receptors and metabotropic P2Y receptors [58,59,60,61,62,63]. The P2X receptors are ligand-gated channels made of proteins with 379–472 amino acids and have two transmembrane domains with a large extracellular loop [64]. These receptors share a trimer topology, and they can assemble as both homomeric and heteromeric trimers of two transmembrane domain subunits that form non-selective cation channels [65]. P2X receptors open in response to micromolar ATP binding, resulting in the flow of cations such as Na^+^, K^+^, and Ca^2+^ across the cell membrane (see review [2]). There are seven P2X receptor subtypes (P2X receptor-1, -2, -3, -4, -5, -6, and -7) expressed in mammalian tissues [56,57]. Specialized functions are achieved by different P2X receptor subtypes in different cell types depending on the subtype expression profile, subcellular distributions, and their biophysical properties [2,56,57].

The P2Y receptors are G-protein coupled receptors, which, in turn, activates intracellular second messenger systems to modulate the physiological function of the cells. In addition to ATP and ADP, P2Y receptors bind to the pyrimidines UTP and UDP. A group of P2Y receptor subtypes (P2Y receptor-1, -2, -4, -6, and -11) are metabotropic receptors mainly coupled with G_q_ proteins to stimulate phospholipase C (PLC)-β followed by inositol 1,4,5-trisphosphate (IP_3_) generation from phosphatidylinositol 4,5-bisphosphate (PIP_2_) and Ca^2+^ mobilization from intracellular stores [63,66]. In particular, the P2Y11 receptor only additionally activate adenylate cyclase [63]. Remaining P2Y receptors, P2Y receptor-12, -13, and -14 are coupled to G_i_ proteins that inhibit adenylate cyclase followed by a reduction of cAMP level in the cytosol [63,67].

Studies have demonstrated several subtypes of P2X receptors in the heart [61,68,69,70]. Early study has shown using immunohistochemical methods that P2X1 receptors in the heart localized to cardiac myocytes [71]. Among the seven subtypes of P2X receptors P2X4 receptors have been shown to be highly expressed in cardiac ventricular myocytes using immunoblotting [72,73] and immunocytochemistry [72,74]. Quantitative polymerase chain reaction (PCR) and in situ hybridization have demonstrated that expression of mRNA of P2X receptors varied in different regions of the heart as well as in different species [75]. In the rat hearts, P2X5 receptor mRNA was the most abundant of the P2X receptors in left ventricle (LV), right atrium and sinoatrial node (SAN) [75]. In human the same methods revealed that mRNA of P2X4 receptor and P2X7 receptor were the highest among P2X receptors in RA cells and SAN [75]. The same method by these authors has shown that, in myocardial infarction (MI)-induced heart failure rats, P2X4 receptor mRNA was up-regulated in the RA cells and SAN [75]. mRNA for P2X1 receptor was specifically expressed in the human SAN, but not in human RA cells [75]. In addition, mRNA for P2X2- and P2X3-receptor and P2Y11 receptor were not detected in human RA cells and SAN [75]. Somewhat consistent observation has been reported in isolated atrial myocytes from rats and mouse atrial cell line HL-1. The levels of P2X4 receptor mRNA have been found to be highest among seven P2X receptor subtypes in these atrial cells [11]. Interestingly, the P2X4 receptor protein level has been shown to be significantly higher in the RA myocytes than LA myocytes from adult rats [11]. P2X5 receptor and P2X7 receptor are also expressed in rat atrial myocytes, and the level of the P2X7 receptor is also higher in the RA myocytes compared with LA myocytes [11]. However, it should be noted that HL-1 cells have more abundant P2X7 receptor proteins compared with intact ventricular and atrial myocytes [11]. This may be because of existence of nodal cells in the HL-1 cell preparation [76] and/or its mouse origin [77]. In mouse atrial cells, P2X7 receptors have been detected and they have been shown to be co-localized with caveolin 1 and 3 [78]. High abundance of P2X7 receptor has also been observed in most of cancer cells [79], which may be one reason for the high P2X7 receptor expression in immortalized HL-1 atrial cells.

The P2X4 receptor is structurally similar to others in the P2X receptor family and binds to ATP with similar EC_50_ to P2X3-, P2X5-, and P2X6-receptors. However, they have higher EC_50_ compared with P2X1- and P2X3-receptors. P2X4 receptor has 1 magnitude lower EC_50_ for ATP compared with P2X7 receptor. Unique property of P2X4 receptor is its resistance to suramin and PPADS, the well-known P2 receptor antagonists [37,54]. In adult ventricular myocytes, 2-methylthio-ATP (2-MeS-ATP), the P2X receptor agonist, causes an increase in a nonselective cation current that is partly resistant to suramin. This current has been shown to be significantly bigger in P2X4 receptor transgenic myocytes [80]. This suramin-resistant current turned out to be mediated by P2X4 receptor [80]. This P2X subtype is selectively potentiated by ivermectin, the P2X4 receptor-specific allosteric enhancer. These pharmacological properties permit distinction of P2X4 receptor from other P2X receptors.

Abundant P2Y receptor subtypes expressed in cardiac tissues include P2Y receptor-1, -2, and -6 (Table 1). P2Y1 receptors are expressed in many types of tissues including heart. Only purines can activate P2Y1 receptors, while UTP and its derivatives are not active at this receptor type [81]. ADP and 2-MeS-ADP are potent full agonist for P2Y1 receptor. P2Y2 receptor is also expressed in a wide variety of tissue including heart and it is activated by UTP and ATP with equal potency and efficacy [82]. However, ATPγS is less potent and α,β-methyl-ATP and 2-MeS-ATP are week partial agonist for P2Y2 receptor [82]. P2Y4 receptor has an agonist selectivity similar to that of P2Y2 receptor. P2Y6 receptor is known to be expressed in the heart and it is activated by UDP and UTP with higher affinity to UDP than UTP, ATP, and ADP [63].

It has been found that there are differences in the expression profiles of P2Y receptor subtypes within the heart and among the species. It has been reported by Musa et al. (2009) [75] that the mRNA level for P2Y receptor-1, -2, and -14 were highest for P2Y receptor in LV, while in rat RA and SAN, P2Y2 receptor and P2Y14 receptor levels are highest. P2Y1- and P2Y2-receptor mRNA have been shown to be abundant for P2Y receptor in the RA, while P2Y1-, 2-, and 14-receptor are abundant P2Y receptor in human SAN [75]. In the adult rat ventricular myocytes, P2Y1-, P2Y2- and P2Y6-receptor mRNA have been detected with higher P2Y1 receptor expression, while in neonatal rat heart, mRNA of P2Y1-, P2Y2-, P2Y4- and P2Y6-receptors have been detected [83]. In the neonatal fibroblast, P2Y1 and P2Y6 appears to be expressed at higher levels than P2Y2- and P2Y4-receptor [83]. The P2Y1 receptor expression and cell membrane immunofluorescence have been found in pacemaker cells of toad hearts [84]. Another paper has reported using the PCR analysis significant expressions of P2Y1-, P2Y2-, and P2Y6-receptors in mouse heart [7], with high abundance of P2Y1- and P2Y6-receptor mRNA. Recently, it has been reported in isolated rat atrial myocytes that LA myocytes have two-fold higher P2Y1 receptor protein levels compared with RA myocytes [11].

## 4. Regulation of Cardiac Contractility by ATP and Roles of P2 Receptors

In the heart, extracellular ATP exerts both negative and positive inotropic effects (For review see [2]). Extracellular ATP changes cardiac contraction biphasically and the effects are different among different species (Table 2). There are some controversies among the observations on the effects of purinergic receptor antagonists on the ATP-induced negative and positive inotropy (Table 2). In rat and human atrium, ATP first decreases contraction, which is followed by a positive inotropic effect [136,137]. It has been demonstrated in electrically driven rat LA tissue that, ATP, ADP, AMP, adenosine and UTP causes a dual inotropic effect: first a rapid decrease in contractility, and second an increase in contractile tension [136]. The P2X receptor agonist 2-MeS-ATP has only induced a negative inotropic effect in the rat LA tissue [136]. The A1 receptor antagonist, 1,3-dipropyl-8-cyclopentylxanthine (DPCPX), has inhibited the negative effects of ATP and adenosine [136]. In contrast, in human cardiac atrium, it has been shown that ATP has biphasic effects like those seen in rat atrium, but that A1 receptor antagonist DPCPX or suramin does not suppress negative inotropy by ATP [137]. PLC blockade has not affected ATP-induced biphasic effects [137]. In this paper, they have shown that 2-MeS-ATP increases contraction and does not induce negative inotropy in human atrium. However, ATPγS has shown biphasic inotropy, which means that the effects are not caused by metabolite of ATP and suggests possible role of P2X receptor in the positive inotropy. UTP, however, induces a positive inotropic effect mediated by suramin-sensitive receptors in human, rat and mouse atrium [136,137,138]. This UTP-induced positive inotropic effect has been suppressed by PLC inhibition (U73122) or protein kinase A inhibition, and suggested to be mediated by P2Y2- or P2Y4-receptors [138].

There are some controversies among the previous reports on the role of P2X receptor on ATP-mediated negative or positive inotropy among the species and heart regions (Table 2). In rat and guinea pig atrium the positive inotropic effect of ATP has been shown to be sensitive to suramin or reactive blue, while the negative inotropic effect of ATP in rat and guinea pig has been blocked by DPCPX [136,139]. In rat P2X receptor agonist 2-MeS-ATP has decreased contraction, but in mouse and chicken cardiac cells it has increased contractility [136]. In rat ventricle it has been shown that ATP only increases contraction via enhancement of Ca^2+^ current and Ca^2+^ transient [140]. The ATP-mediated positive inotropic effect in cardiac muscle is also mediated by cytosolic alkalinization, but not by sensitization of myofilament [149,150]. In human atrium, the positive inotropic effect of ATP has been suggested to be mediated by P2X4-like receptors because it was not blocked by suramin, the non-specific P2 receptor antagonist, or by PLC blocker or adenylate cyclase inhibitor [137]. In mice ventricular myocytes, evidence has further shown a role of P2X4 receptor on ATP-induced positive inotropy. In this regard, treatment of P2X agonist (2-MeS-ATP) or ivermectin has increased cell shortening in mice ventricle cells [141]. In addition, these agonists failed to show positive inotropy in P2X4 receptor knock-out mouse cardiac cells. In human P2X4 receptor-overexpressed mice ventricular myocytes, 2-MeS-ATP induced greater increase of myocyte contraction than in wild-type myocytes [72]. Consistently, in cardiac myocytes from cardiac-specific P2X4 receptor overexpression showed mild enhancement of cardiac contractility without having hypertrophy or cardiomyopathy [72].

## 5. Regulation of Heart Rate by P2 Receptors

In clinic, ATP is used to treat supraventricular arrhythmias mainly in children, because adenosine degraded from ATP in serum activates P1 receptors [151]. In frog atria, it has been also shown that the P1 agonist adenosine mimicked the negative chronotropic effect of ATP [61]. In mammalian SAN cells, adenosine activates acetylcholine-activated K^+^ channels (K_ACh_) [152,153]. However, in toad pacemaker cells, adenosine (1–1000 μM) has not shown any effect on either firing rate or intracellular Ca^2+^ concentrations. In these cells, Ju et al. (2003) [84] have shown that ATP (100 μM) application still transiently increases beating rate and Ca^2+^ transient amplitudes, which is followed by decrease in the rate of beating [84]. They also have shown that this effect is well-mimicked by P2Y1 receptor agonist 2-MeS-ADP (1–5 μM), but not by P2X1- or -3 receptor agonist (α,β-mATP), and that it is suppressed by P2Y1 receptor inhibitor, the bisphosphate derivative, 2’-deoxy-N6-methyladenosine-3’,5’-bisphosphate (MRS 2179) [154] or by the PLC inhibitor (U73122). The large Ca^2+^ increase by 2-MeS-ADP in toad SAN cells seems to be similar to caffeine-induced Ca^2+^ release. The secondary negative chronotropy by ATP in these cells has been suggested to be associated with partial sarcoplasmic reticulum (SR) Ca^2+^ depletion [84].

In SAN-containing beating atrial strip from rat, ATP has decreased heart rate and contractility [142]. The negative chronotropy induced by ATP in this preparation has been suggested to be due to activation of P2X4 receptors [142]. Such role of P2X4 receptor in the ATP-mediated negative chronotropy has also been suggested by other group [72]. The proposed functions of P2X4 receptors, negative chronotropy and positive inotropy, are thought to be somewhat similar to the negative chronotropy and positive inotropy by digitalis [155,156]. Electrophysiological investigation in HEK293 and *Xenopus* oocytes have provided evidence that activation of P2X4 receptors leads to permeation of various cations (mainly Na^+^) through the cell membrane [157,158]. Therefore, inhibition of Na^+^-Ca^2+^ exchanger (NCX) by activation of P2X4 receptor similar to the action of digitalis via Na^+^-K^+^ pump inhibition has been suggested to suppress SAN beating rate [142]. Further electrophysiological investigation on this mechanism involving crosstalk between P2X4 receptor and NCX in the heart warrants further investigations.

## 6. Role of P2 Receptors in Cardiac Stress Responses

Extracellular ATP has been thought to have beneficial effects on the heart via its metabolic product adenosine [2,159,160]. However, a line of evidence also suggests that ATP itself exerts cardioprotective effects via P2X4 receptors (Table 2). Cardiac overexpression of P2X4 receptor does not produce any hypertrophy or failure, but it only modestly increases basal cardiac contraction [72]. However, enhanced in vivo contractility is not associated with enhanced contraction in single cardiac myocytes, supporting the notion that extracellular ATP activates overexpressed P2X4 receptor to induce an increased in vivo contractile function [72]. In P2X4 receptor overexpressing cardiomyocyte the P2X4 receptor agonist has enhanced contraction, but has not modulated L-type Ca^2+^ channel [161]. It has been suggested that the entry of Na^+^ through P2X4 receptors can increase intracellular Ca^2+^ concentration via affecting the NCX [161]. In fact, P2X receptor agonist increases Ca^2+^ transient and SR Ca^2+^ loading, of which effects were larger in P2X4 receptor transgenic myocytes, providing a mechanism for P2X4 receptor-mediated increase in contractility in this mice ventricle [161].

It has been shown that the P2X4 receptor knock-out mice develop worse heart failure phenotype after coronary artery ligation or pressure overload by transverse aortic constriction, such that it depresses contractile function faster and more significantly in pressure overload or MI-induced heart failure in mice [141]. The cardioprotective role of P2X4 receptors has been thought to be partly mediated by endothelial NO synthase (eNOS). In fact, P2X4 receptors are co-immunoprecipitated and colocalized with eNOS in mouse ventricular myocytes [141]. Cardiac specific overexpression of P2X4 receptors in cardiac myocytes increased S-nitrosylation, cGMP, and NO formation, and protected heart from pressure overload and infarction induced heart failure [141].

Another pathway of beneficial effect exerted by ATP itself is P2X7 receptor. It has been reported that activation of P2X7 receptors by ATP can also protect cardiac muscle under ischemia-reperfusion injury in the heart. The cardiac ischemia-reperfusion injury is prevented with appropriate treatments initiated either before (preconditioning) or immediately after (postconditioning) the index ischemia. Ischemia preconditioning or postconditioning has been shown to induce release of endogenous cardioprotectants from cardiomyocytes via the opening of a channel formed by the interaction of a P2X7 receptor with a pannexin 1 hemichannel [143,162]. It has been demonstrated that P2X7 receptor opening by ATP makes coupling between P2X7 receptor and pannexin-1, thereby opening the pannexin-1 [163]. In fact, ATP is released from ischemic cardiac tissues, and ATP as well as P2X7 receptor agonist (benzoyl benzoyl-ATP, BzATP) has also been suggested as cardioprotectants to activate this pathway [144]. Taken together, one may think that these effects by ATP though P2X receptors (P2X4- or P2X7-receptor) may involve a crosstalk between ATP release pathway and P2X receptors in a microdomain since ATP can be easily broken down by enzymes once they are released from cells.

In mouse atrial myocytes, it has been shown that caveolin 1 and 3 are co-localized with PX7 receptors [78]. The absence of any component of the caveolin and PX7 receptor complex in these preparations has caused compensatory up-regulation of PX7 receptor or caveolins [78]. The complex of PX7 receptors and caveolins are predominantly localized in buoyant membrane fractions (lipid rafts/caveolae) prepared from hearts using detergent-free sucrose gradient centrifugation. It has been shown that the PX7 receptor can accelerate caspase-1 activation [164]. In fact, caspase-1 acts as a potent proapoptotic caspase in isolated cardiac myocytes [165]. Since caveolins may be binding partners for intracellular caspases [166], it may be possible that PX7 receptor regulates inflammation in the heart. However, the link between caveolins and PX7 receptors and its role in caspase activation and inflammation in the heart remain unknown.

Prolonged exposure of cardiac muscle to neurohumoral factors, such as norepinephrine and endothelin-1, induces muscle hypertrophy [167,168,169]. However, extracellular ATP does not seem to induce ventricular hypertrophy, since it has not caused hypertrophy in neonatal ventricular myocytes [145]. UTP, on the other hand, produces cardiac myocyte hypertrophy [145], suggesting a role of P2Y receptors in this muscle remodeling. Consistently, it has also been shown that ATP inhibits norepinephrine- or phenylephrine-induced increase in the size of neonatal ventricular myocytes, and that it reduces hypertrophy marker gene (ex. *ANP*, *MLC-2*) expression in the norepinephrine- or phenylephrine-treated cells [170]. The UTP-dependent hypertrophy in neonatal cardiac myocytes has been shown to be mediated by ERK activation [145]. Interestingly, however, in adult atrial HL-1 cell line, there is an evidence that ATP induces hypertrophic growth similar to endothelin-1 or norepinephrine [9]. In addition, this ATP effect has been suggested to be mediated by type 1 IP_3_Rs localized in the perinuclear region [9]. In fact, the IP_3_R1 is not expressed in ventricular myocytes. These previous findings suggest distinct ATP signaling and/or receptor context and distinct role of ATP in hypertrophic growth of atrial and ventricular myocytes.

It has been suggested that inhibition of P2Y2 receptors may diminish fibrotic remodeling and turnover of extracellular matrix in the heart, because the nucleotide, UTP, induces a profibrotic response via P2Y2 receptor in cardiac fibroblast [146]. P2Y6 receptor-Gα_12,13_ signaling has been shown to mediate pressure-overload induced cardiac fibrosis [7]. Transgenic expression of inhibitory polypeptides of the heterotrimeric G_12_ family G protein (Gα_12/13_) in cardiomyocytes suppressed pressure overload-induced fibrosis without affecting hypertrophy. The mRNA for P2Y6 receptors increases in pressure-overloaded mice having decreased ejection fraction and Gα_12,13_ signaling [7]. This signaling is thought to be associated with cardiac fibrosis, not hypertrophy, and associated with Gα_13_ and stimulated by upstream ATP and UTP releases through pannexin-1 in this pressure-overloaded mice ventricles [7]. Regarding the role of P2Y6 receptor in the cardiac pathogenesis the previous reports have shown contradictory findings. Deletion of P2Y6 receptor, in fact, promoted pressure overload-induced sudden death, as well as cardiac remodeling and dysfunction. Mice with cardiomyocyte-specific overexpression of P2Y6 receptor also exhibited cardiac dysfunction and severe fibrosis. In contrast, P2Y6 receptor deletion had little impact on oxidative stress-mediated cardiac dysfunction induced by doxorubicin treatment [171].

Pressure overload and volume overload in the heart are associated with cardiac myocyte remodeling and dysfunction, leading to arrhythmogeneis and failure. Such mechanical forces are clinically related to hypertension, heart failure, and valvular heart diseases and include stretch, shear stress, and afterload increase. Enlarged cardiac chamber has been observed under heart failure and stretch signaling has been thought to play an important role in the pathogenesis of such congestive heart failure and subsequent arrhythmias [172,173]. Stretch and shear stress can induce ATP release from cardiac myocytes, and therefore, they could activate P2 receptors. Role of P2 receptors in endothelial cell shear stress responses has been relatively well understood. For example, endothelial P2X4 receptor channels are crucial to flow-sensitive mechanisms that regulate blood pressure and vascular remodeling. It has been shown by Yamamoto et al. (2006) [147] that P2X4 receptor knock-out mice have higher blood pressure and do not have normal endothelial cell responses to flow, such as influx of Ca^2+^ and subsequent production of the potent vasodilator NO. Blood vessel dilation induced by acute increases in blood flow is markedly suppressed in P2X4 receptor knock-out mice.

**Figure 1:**
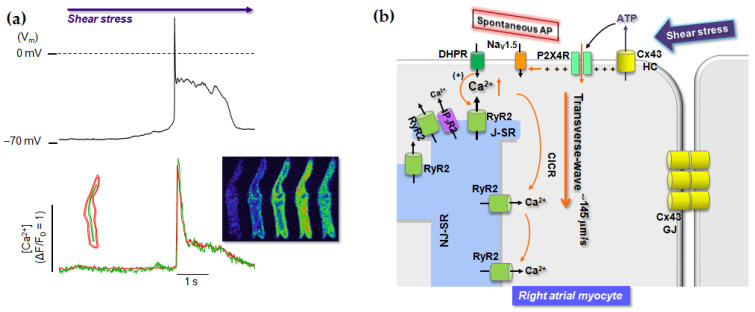
Role of P2X4 receptor in shear stress-induced action potential in rat cardiac atrial myocytes. (**a**), shear stress-induced atrial action potential (upper) and Ca^2+^ releases (lower) simultaneously measured in a right atrial (RA) myocyte from rat. Red and green traces represent peripheral and central Ca^2+^ signals, respectively, measured from ROI shown in the left side of traces, showing transverse Ca^2+^ wave (see confocal Ca^2+^ images on the right side). (modified from [9]) (**b**), Schematic diagram representing hypothetical shear stress signaling pathway associated with transverse Ca^2+^ wave. The signaling is known to start from the same ATP release via connexin 43 (Cx43) hemichannels (HC) and subsequent activation of P2X4 receptor (P2XR). Cation influx though P2X4 receptor channel can depolarize membrane potential to trigger spontaneous action potential and secondary transverse Ca^2+^ wave. (modified from [11]).

**Figure 2:**
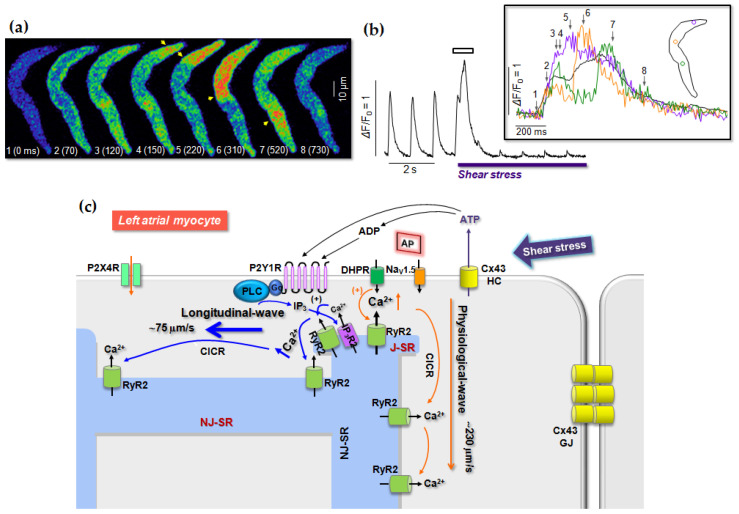
Role of P2Y1 receptor in shear stress-mediated longitudinal Ca^2+^ wave in rat cardiac atrial myocytes. (**a**), Alteration of Ca^2+^ transients by shear-induced longitudinal Ca^2+^ wave. Left images show shear stress (16 dyn/cm^2^)-induced longitudinal Ca^2+^ propagation (arrowheads) during regular Ca^2+^ signaling in a representative rat left atrial (LA) myocyte. (**b**), Ca^2+^ signal measured from confocal Ca^2+^ images in the cell shown in the panel A. Shear stress-induced longitudinal Ca^2+^ wave enhances Ca^2+^ release on depolarization, which is soon followed by dramatic reduction in the regular Ca^2+^ transients (modified from [148]). Inset shows local Ca^2+^ signals measured from region-of-interest (ROI) marked by the same color. (**c**), Schematic diagram showing hypothetical model of shear stress-induced signaling pathway for the generation of longitudinal Ca^2+^ wave in most of LA cells. Under shear stress ATP is first released from Cx43 hemichannel (HC), which, in turn, triggers P2Y1 receptor-IP_3_R signaling to trigger the Ca^2+^ wave. Ca^2+^-induced Ca^2+^ release (CICR) occurs via IP_3_R and ryanodine receptor type 2 (RyR2) crosstalk via Ca^2+^. This shear-induced Ca^2+^ wave (~75 μm/s; [11]) is different from physiologically occurring action potential (AP)-induced transverse Ca^2+^ wave (~230 μm/s; [175]) in terms of direction and speed of Ca^2+^ movement. (modified from [11]).

Role of P2 receptors in cardiac myocytes in mechanotransduction and their involvement in cardiac cell pathogenesis, however, are poorly understood. In this regard, there are recent evidence on the role of P2 receptors in shear stress-induced two different types of global Ca^2+^ waves. In fact, the same shear force elicits action potential (AP)-involved transverse Ca^2+^ wave in most of RA myocytes, but it induces slow longitudinal Ca^2+^ wave in a majority of LA myocytes [11,174]. Interestingly, the different types of shear-stress-induced Ca^2+^ waves in atrial myocytes have been discovered to be dependent on distinctly distributed P2 receptor subtypes between LA myocytes and RA myocytes [11]. In this regard, shear stress-induced spontaneous action potential in RA myocytes has been suppressed by specific inhibition of P2X4 receptors ([9]; Figure 1), while longitudinal Ca^2+^ wave in LA myocytes under shear stress has been known to be due to activation of P2Y1 receptor-PLC-IP_3_R type 2 signaling with subsequent Ca^2+^-induced Ca^2+^ release via ryanodine receptor type 2 (RyR2) ([174]; Figure 2). Consistently, higher P2Y1 receptor levels in LA myocytes than RA myocytes and more abundance of P2X4 receptors in RA myocytes versus LA myocytes have been demonstrated [11]. The P2Y1 receptor-mediated slow Ca^2+^ wave propagation can disturb normal Ca^2+^ signaling having Ca^2+^ propagation in a transverse direction ([148]; Figure 2). Note that atrial cells lack transverse-tubules [175,176], such that action potential triggers L-type Ca^2+^ current-induced Ca^2+^ release in the peripheral domain first. This peripheral Ca^2+^ increase, then, propagates into the cell interior via diffusion-dependent sequential RyR2 activations in a transverse direction [148,177,178,179]. Finally, the shear-induced P2Y1 receptor signaling results in significant attenuation of regular Ca^2+^ transients ([174]; Figure 2b), thereby causing LA contractile dysfunction. This can increase thrombus formation in atrial chamber and decrease of ventricular ejection. Shear stress-mediated P2X4 receptor signaling in resting atrial cells is associated with AP generation and depolarization ([9]; Figure 1), which may also alter rhythmic Ca^2+^ release process mostly in RA cells. Specific roles of P2X4 receptors and P2Y1 receptors in atrial myocytes under shear stress and their role in volume- or pressure-overload-mediated atrial remodeling and arrhythmogenesis remain to be uncovered.

## 7. Concluding Remarks

Cardiac myocytes express several types of P2X- and P2Y-receptor subtypes and themselves release ATP under various stimuli. So far, the effects of ATP or UTP on cardiac contraction and rhythm have been studied. There is some consensus on the role of P2X4 receptors in positive inotropy in ventricular tissue and negative chronotropy in beating atrial tissue based on a line of evidence with pharmacological and genetic interventions. A role of P2Y1 receptor subtype, one abundant P2Y receptor in cardiac myocytes, has been found in the positive regualtion of rhythm in SAN preparation and in pathologic alteration in LA cell Ca^2+^ signaling with shear stress. UTP signaling appears to be involved in cardiac remodeling and fibrosis via P2Y6 receptor, and also modulate atrial contraction although there are still contradictory findings with regard to P2Y6 receptor. Accumulated findings strongly suggest that ATP release and subsequent activation of P2 receptors are major signaling pathway activated by mechanical stimulus in the ventricular and atrial muscles, and that different responses between differnt sides of cardiac chamber can be achived by adopting distict P2X- or P2Y-receptor subtypes. In the pathologic hearts a signaling between ATP release pathway and purinergic receptor activation may occur in a compartmentalized microdomains in cardiac myocytes more significantly because of larger mechanical stresses and they could play a role in myocytes remodeling, functional alterations, and fibrosis. Role of P2 receptor subtypes in different cardiac pathogenesis with distict environmental changes needs to be further discovered considering cardiac regions.

## Figures and Tables

**Table 1:** P2Y-receptor subtypes expressed in mammalian tissues and their agonist affinities.

Type	Species	Principal Agonists	Tissue Distribution	Selected References
P2Y1	Rat	2-MeS-ADP = 2-MeS-ATP > ADP	Heart, platelet, skeletal muscle, neuron, intestine	[85,86]
	Mouse	2-MeS-ATP > 2Cl-ATP > ATP		[85]
	Bovine	2-MeS-ATP > ADP > ATP		[87]
	Human	(N)-mc-2-MeS-ADP > 2-MeS-ADP > ADP = ADPβS ≫ ATP		[66,88,89,90,91,92,93]
P2Y2	Rat	UTP = ATP > CTP > GTP	Heart, lung, skeletal muscle, spleen, kidney	[94,95,96]
	Mouse	UTP = ATP> Ap4A		[82]
	Canine	UTP ≥ ATP > ADP > 2-MeS-ATP		[97]
	Porcine	UTP > ITP > ATP > UDP		[98]
	Human	UTP = ATP > INS37217 > Ap4A > ATPγS		[99,100,101,102]
P2Y4	Rat	UTP = ATP = ITP = Ap4A	Placenta, lung, vascular smooth muscle, brain, liver	[96,103,104,105]
	Mouse	UTP = ATP		[106]
	Human	UTP > UTPγS		[101,107,108,109,110]
P2Y6	Rat	UDP > UTP > ADP > 2-MeS-ATP	Heart, lung, spleen, placenta, thymus, intestine, brain	[101,111]
	Mouse	UDP > UTP > ADP > 2-MeS-ATP		[112]
	Human	UDP = 5-Br-UDP >> UTP > 2-MeS-ADP		[113,114,115]
P2Y11	Canine	ADPβS = 2-MeS-ADP ≥ 2-MeS-ATP > ATP	Spleen, intestine, immune cells	[116,117]
	Human	ARC67085 ≥ ATPγS = BzATP > ATP, (UTP) > 2-MeSAT		[116,117,118,119,120]
P2Y12	Rat	2-MeSADP >ADP > ATP	Neuron, platelet	[121,122]
	Mouse	2-MeSADP >ADP > ADPβS		[123,124,125]
	Bovine	2-MeS-ADP ≫ ADP, ATP		[126]
	Human	2-MeS-ADP >ADP >> (N)-mc-2-MeS-ADP		[121,127,128]
P2Y13	Rat	ADP > 2-MeS-ADP >> HATP	Spleen, leucocytes, bone marrow, liver, brain	[129]
	Mouse	ADP = 2-MeS-ADP = ADPβS > ATP		[130]
	Human	2-MeS-ADP > (=) ADP > ADPβS		[130,131,132]
P2Y14	Rat	UDP-glucose	Placenta, adipose tissue, intestine, brain, spleen	[133]
	Mouse	UDP-glucose		[134]
	Human	UDP-glucose > UDP-galactose		[135]

ARC67085, 2-propylthio-β,γ-dichloromethylene-D-ATP; Ap4A, diadenosine-tetraphosphate; ATPγS, adenosine-(O-3-thiotriphosphate); 5-Br-UDP, 5-bromo-UDP; BzATP, benzoyl–benzoyl–ATP; 2-Cl-ATP, 2-chloro-ATP; INS37217, P^1^-(uridine 5′)-P^4^-(2′-deoxycytidine-5′)tetraphosphate; 2-MeSADP, 2-methylthio-ADP; (*N*)-mc-2-MeSADP, (*N*)-methanocarba-2-methylthio-ADP (= MRS2365); 2-MeSATP, 2-methylthio-ATP; UTPγS, uridine-(O-3-thiotriphosphate).

**Table 2:** Pathophysiological functions of P2 receptor subtypes in cardiac cells.

Function	Cardiac Regions	Agonist	Effect	R	Species	References
Contraction	LA	ATP, ADP, UTP, Adenosine 2-MeS-ATP	Biphasic(Neg-pos) Neg Neg	A1	Rat	[136]
	A	ATP, ATPγS 2-MeS-ATP	Biphasic (neg-pos) Pos	P2X4(?)	Human	[137]
	A	UTP	Pos	P2Y	Human, rat, mouse	[136,137,138]
	A	ATP	Biphasic (pos-neg)	P2-A1	Rat, guinea-pig	[136,139]
	A	2-MeS-ATP	Neg	P2X	Rat	[136]
	A	2-MeS-ATP	Pos		Mouse, chicken	[136]
	V V	ATP 2-MeS-ATP, ivermectin	Pos Pos	P2X4	Rat Mouse	[140] [72,141]
Heart rate	A SAN RA	Adenosine ATP, 2-MeS-ADP ATP	Neg Biphasic (pos-neg) Neg	P2Y1-SR Ca^2+^ (↓) P2X4	Frog Toad Rat	[61] [84] [72,142]
Pathology	V V V V A(HL-1) Fiboblast V V	ATP UTP ATP	Anti-HF IR-injury (↓) Inflammation (?) Hypertrophy Hypertrophy Fibrosis Fibrosis Antihypertensive	P2X4 P2X7 P2X7 P2Y P2Y(?) P2Y2 P2Y6 P2X4	Mouse Rat Mouse Rat Mouse Human Mouse Mouse	[141] [143,144] [78] [145] [9] [146] [7] [147]
Mechano- transduction	RA	ATP	Shear stress, depolarization	P2X4	Rat	[9,11]
	LA	ATP	Shear stress, Ca^2+^ dysregulation	P2Y1	Rat	[11,148]
	Endo	ATP	Shear stress	P2X4	Mouse	[147]

A, atrium; Endo, endothelium; HF, heart failure; HL-1, HL-1 cells; IR, ischemia-reperfusion; LA, left atrium; neg, negative (: decrease, ↓); pos, positive (: increase); R, receptor; RA, right atrium; SAN, sino atrial node; V, ventricle; (?), hypothesis.

## Data Availability

Data available in a publicly accessible repository.

## Abbreviations

2-Cl-ATP	2-chloro-ATP
2-MeS-ADP	2-methylthio-ADP
2-MeS-ATP	2-methylthio-ATP
5-Br-UDP	5-bromo-UDP
ADP	Adenosine diphosphate
AMP	Adenosine monophosphate
ANP	Atrial natriuretic peptide
AP	Action potential
Ap4A	Diadenosine tetraphosphate
ARC67085	2-propylthio-β,γ-dichloromethylene-D-ATP
ATP	Adenosine triphosphate
ATPγS	Adenosine-(O-3-thiotriphosphate)
BzATP	Benzoyl–benzoyl–ATP
cAMP	Cyclic AMP
cGMP	Cyclic guanosine monophosphate
CICR	Ca^2+^-induced Ca^2+^ release
Cx43	Connexin 43
DPCPX	1,3-dipropyl-8-cyclopentylxanthine
eNOS	Endothelial nitric oxide synthase
ERK	Extracellular signal-regulated kinase
INS37217	P1-(uridine 5′)-P4-(2′-deoxycytidine-5′)-tetraphosphate
IP_3_R	Inositol 1,4,5-trisphosphate receptor
K_Ach_	Acetylcholine activated K^+^ channels
LA	Left atrial
LV	Left ventricle
MI	Myocardial infarction
MLC-2	Myosin light chain-2
MRS 2179	2’-deoxy-N6-methyladenosine-3’,5’-bisphosphate
(N)-mc-2-MeSADP	(N)-methanocarba-2-methylthio-ADP
NO	Nitric oxide
PCR	Polymerase chain reaction
PLC	Phospholipase C
PPADS	Pyridoxal phosphate-6-azo(bensene-2,4-disulfonic acid) tetrasodium
RA	Right atrial
ROI	Region-of-interest
RyR2	Ryanodine receptor type 2
SAN	Sinoatrial node
SR	Sarcoplasmic reticulum
UDP	Uridine diphosphate
UTP	Uridine triphosphate
UTPγS	Uridine-(O-3-thiotriphosphate)
